# Comparison of two oral probiotic preparations in a randomized crossover trial highlights a potentially beneficial effect of *Lactobacillus paracasei* NCC2461 in patients with allergic rhinitis

**DOI:** 10.1186/2045-7022-4-1

**Published:** 2014-01-06

**Authors:** Yannick Perrin, Sophie Nutten, Régine Audran, Bernard Berger, Rodrigo Bibiloni, Jacqueline Wassenberg, Nathalie Barbier, Vincent Aubert, Julie Moulin, Anurag Singh, Corinne Magliola, Annick Mercenier, François Spertini

**Affiliations:** 1 Division of Immunology and Allergy, Centre Hospitalier Universitaire Vaudois, Lausanne, Switzerland; 2 Nestlé Research Centre, Lausanne, Switzerland; 3 Current address: AgResearch Ltd, Food and Bio-based Products, Hamilton, New Zealand

**Keywords:** Allergic rhinitis, Immunomodulation, Nasal provocation test, *Lactobacillus paracasei*, Clinical trial, Grass pollen

## Abstract

**Background:**

There is promising but conflicting evidence to recommend the addition of probiotics to foods for prevention and treatment of allergy. Based on previous studies with fermented milk containing *Lactobacillus paracasei* NCC2461, we aimed to compare the effect of a powder form of the latter probiotic with the effect of a blend of *Lactobacillus acidophilus* ATCC SD5221 and *Bifidobacterium lactis* ATCC SD5219 in patients with allergic rhinitis.

**Methods:**

A double-blind, randomized, cross-over study, involving 31 adults with allergic rhinitis to grass pollen, was performed outside the grass pollen season (registration number: NCT01233154). Subjects received each product for 4-weeks in two phases separated by a wash-out period of 6 to 8 weeks. A nasal provocation test was performed before and after each 4-week product intake period, and outcome parameters (objective and subjective clinical symptoms; immune parameters) were measured during and/or 24 hours after the test.

**Results:**

Out of the 31 subject enrolled, 28 completed the study. While no effect was observed on nasal congestion (primary outcome), treatment with NCC2461 significantly decreased nasal pruritus (determined by VAS), and leukocytes in nasal fluid samples, enhanced IL-5, IL-13 and IL-10 production by peripheral blood mononuclear cells in an allergen specific manner and tended to decrease IL-5 secretion in nasal fluid, in contrast to treatment with the blend of *L. acidophilus* and *B. lactis*.

**Conclusions:**

Despite short-term consumption, NCC2461 was able to reduce subjective nasal pruritus while not affecting nasal congestion in adults suffering from grass pollen allergic rhinitis. The associated decrease in nasal fluid leukocytes and IL-5 secretion, and the enhanced IL-10 secretion in an allergen specific manner may partly explain the decrease in nasal pruritus. However, somewhat unexpected systemic immune changes were also noted. These data support the study of NCC2461 consumption in a seasonal clinical trial to further demonstrate its potentially beneficial effect.

## Introduction

Current available treatments for allergic rhinitis are not devoid of side-effects and primary prevention strategies are non-existent. This has led to the search for new approaches to prevent or reduce allergic symptoms and improve quality of life of patients. In this context, and regarding its safety profile, interest in nutritional intervention for allergy management has been growing. Recent scientific evidence from pre-clinical studies and human clinical trials has highlighted different nutritional interventions such as vitamins, lipids, dietary polyphenols and probiotics as promising agents that can impact both allergic sensitization and alleviate allergic symptoms [1-3]*.* Probiotics are defined as “live microorganisms which when administered in adequate amounts confer a health benefit on the host” [4]. The “hygiene hypothesis” suggested that modern living conditions may have affected the initial establishment of the intestinal microbiota, with subsequent impact on the development of the GALT (gut-associated lymphoid tissue) at an early age, and therefore on the regulation of local and systemic immune responses [5]. In addition, several studies pointed out differences in the gut microbial composition between infants developing allergy or not, suggesting a crucial role of the intestinal microbiota on the immune system orientation [6-9].

Most clinical studies in the field of probiotics and allergy have focused on the prevention or treatment of atopic dermatitis, with divergent outcomes likely resulting from different probiotic strains, target populations, and study designs [10-15]. Although evidence of a beneficial effect of probiotics on allergic respiratory symptoms is still conflicting [16,17], a few studies conducted in children and adults with allergic rhinitis suggested a beneficial effect of the consumption of specific probiotic strains belonging to the *Lactobacillus casei*, *Bifidobacterium longum*, *Bifidobacterium lactis* or *Lactobacillus paracasei* species [18-22]. We also recently showed in a crossover, placebo controlled clinical trial that the administration of a fermented milk containing *Lactobacillus paracasei* NCC2461 yielded encouraging results on allergic rhinitis symptoms and immune biomarkers [23]. To reinforce our previous observations on *Lactobacillus paracasei* NCC2461, we conducted the present study to compare the effect of a 4-week consumption of two probiotic preparations on clinical and biological responses to a well-reproducible nasal provocation test (NPT) with grass pollen, in adult volunteers with grass pollen allergic rhinitis. The second probiotic preparation (combination of *L. acidophilus* SD5221 and *B. lactis* SD5219) was chosen based on promising results obtained in children with birch pollen allergy [24].

## Material and methods

### Population

**Figure 1:**
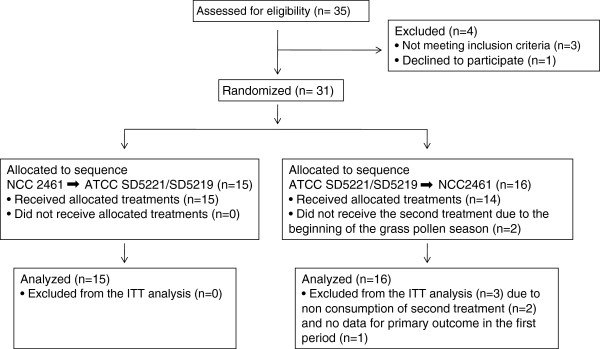
Participants’ recruitment and inclusion flow chart.

Thirty-one adult volunteers with a history of grass pollen allergic rhinitis were enrolled (Figure 1) in a single center study (CHUV, Lausanne, Switzerland) on the basis of the following inclusion criteria: (1) age between 18 and 35 years; (2) a history of allergic rhinitis during the latest grass pollen season confirmed by positive skin prick test (SPT) to grass pollen (wheal diameter > 3 mm); and (3) a positive response to a NPT with grass pollen (combined nasal reaction threshold ≤ 10′000 standardized quality units (SQs)/ml grass pollen or less at the screening/inclusion phase). Volunteers were excluded from the study when presenting any medical condition that could influence the study (pregnancy, viral or bacterial airway infection, active allergic rhinitis), or uncontrolled asthma (peak expiratory flow < 20% of volunteer’s best personal value), when treated with antihistamine or antibiotics less than two weeks before enrolment or during the trial. Furthermore they were also excluded when presenting with allergic rhinitis to tree pollen or perennial allergic rhinitis. Clinical research protocol was approved by the Ethical Review Board of the Faculty of Biology and Medicine, Lausanne (N° 153/07) as well as by Swiss Regulatory Authorities (Swissmedic, Bern) and all enrolled subjects provided informed consent before the start of the study.

### Study products

Powder sachets contained maltodextrin and probiotics (≈ 10^10^ colony forming unit (cfu) each), either *L. paracasei* NCC2461 (CNCM I-2116; ST11; Nestlé, Switzerland) or a blend (1:3) of *L. acidophilus* ATCC SD5221 and *B. lactis* ATCC SD5219 (Danisco Cultures, USA). They were kept at 4°C until consumption. The 31 subjects were randomized according to a computer generated allocation schedule to receive one of the individually coloured test products and instructed to dilute once a day the content of one sachet into 2 dl of cold milk just before consumption on an empty stomach. Quality control of the powder sachets was carried out for product release, in the middle, and at the end of the study, to ensure viability of the tested probiotics.

### Study protocol

**Figure 2:**
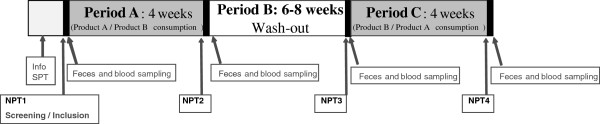
**Study design.** NPT: nasal provocation test; SPT: skin prick test; Info: information to subject.

The study was designed as a randomized, double-blind, two-treatment, cross-over clinical trial, performed outside of the grass pollen season (between August 2007 and March 2008; registration number: NCT01233154) to evaluate the effect of a 4-week consumption of two probiotic formulations (period A and period C) on clinical and biological responses to NPT (Figure 2). Both investigators and subjects were aware that the aim of the study was to test two different probiotic preparations, but they were both blinded with regards to the probiotic preparation. The choice of the duration (four weeks) of the treatment period was mainly based on the encouraging results obtained with *L. paracasei* NCC2461 in a previous clinical trial [23]. Products were supplied before each period of product consumption. Both treatment periods were separated by a wash-out period (period B) of six to eight weeks. A NPT was performed before and after each treatment period, and outcome parameters were measured during and/or 24 hours after each NPT. Non solicited adverse events were recorded all along the trial. Compliance was evaluated on subjects’ product consumption reported in diary cards and from stool samples analysis of studied probiotics at the beginning and the end of each treatment period.

The primary outcome of the study was defined as the difference before and after treatment with each probiotic preparation on subjective nasal congestion by visual analogical scale (VAS). We additionally compared the two treatments in their capacity to modify this endpoint. Secondary endpoints included 1) differences before and after treatment in allergen dose levels necessary to reach the combined reaction threshold. Each clinical criterion and their respective thresholds were also examined separately as secondary endpoints and compared between the two treatment arms; 2) nasal pruritus evaluated by VAS as well as immune markers from blood and nasal samples as described below.

### NPT and clinical assessment

The NPT was performed as described previously [25-27], before and after each 4-week product intake period. The “combined nasal reaction threshold” was defined as the dose level of the allergen extract for which at least two of the three following clinical criteria were fulfilled: (1) ≥ 5 sneezes in first ten minutes after challenge; (2) ≥ 0.5 g nasal secretion in the first ten minutes after challenge (above diluent value); (3) a decrease in peak nasal inspiratory flow (PNIF) ≥ 40% or minimal cross-sectional area (MCA) ≥ 30% of baseline (diluent value), whichever came first [28].

### Nasal samples and immune markers

Nasal samples were collected before NPT, 10 min. after the 1′000 SQ/ml challenge and 24 h after NPT: cells were obtained by scraping the inferior turbinate and nasal septum of a single nostril three times with an endocervical sampling brush (cyto-brosse, Charles Berdat, Switzerland) as previously described [19], and fluids by application of 2 filter paper strips (6×30 mm, Whatman N° 903, Whatman Paper Ltd., Maidstone, UK) in the controlateral nostril (inferior turbinate and nasal septum) for 10 min [29]. After removal, the strips were placed in a 2 ml silicone-coated tube in dry-ice and stored at −80°C until processed. Papers were eluted in 1 ml of Tris buffer 0.1 M, pH 7.4, 0.002% Tween 20 by strong agitation at 4°C during at least 2 h [30]. Cytokines (IL-5 and TNFα) were measured in nasal fluid by multiplexed bead-based flow cytometry (Fluorokine multiplex kit, R&D Systems, Inc, Minneapolis, USA) using a Luminex 100 analyser (detection limits: 0.21 and 0.59 pg/ml respectively) [23].

### Blood samples and immune markers

Serum samples (10 ml) for measuring specific IgE and IgG4 levels to grass pollen, were collected 24 h after NPT, before and after each product consumption period (Figure 2) and stored at −80°C until analysis. Specific IgG4 and IgE were measured by the CAP System (UniCAP 100, Pharmacia Diagnostics, Uppsala, Sweden). For the in vitro stimulation of peripheral blood mononuclear cells (PBMCs), whole blood was collected 24 h after NPT (before and after each product consumption period), in sodium citrate tubes (Vacutainer® CPT™, BD, Basel, Switzerland), and PBMCs purified by centrifugation over a density gradient. After washing, PBMCs were stored in liquid nitrogen until use. PBMCs were thawed, and cultured at 2×10^6^ cell/ml as previously described [23] in the presence of 90 μg/ml of grass pollen extract (ALK Wässrig SQ; ALK-Abello, Hørsholm, DK), or a cocktail of vaccine antigens (5 μl/ml Candida mannan (NIBSC, London, UK), 10 μg/ml tetanus toxoid, 5 μg/ml Tuberculin Purified Protein derivative (PPD, SSI, Copenhagen, DK)), or medium alone. The supernatants were collected after 6 days of culture, and stored at −20°C until evaluation of their cytokine contents (IL-5, IL-8, IL-10 and IFNγ by multiplex flow cytometry as described above and IL-13 by ELISA (Diaclone, Besançon, F); detection limits: 0.21, 0.50, 0.34, 1.28 and 0.8 pg/ml respectively).

### Fecal samples

Fecal samples were collected as described in Figure 2 into sterile tubes maintained in anaerobic conditions using the AnaeroGen system (Oxoid, UK) and kept at <10°C until processing within 12 hours. Briefly, 100 mg of faeces sample were resuspended in sterile saline and serial dilutions performed (10^-2^-10^-6^). Then, 100 μL of each dilution were plated on MRS and BSM media [31]. Plates were incubated at 37°C in anaerobic conditions. After 48 h, the whole bacteria lawn was scraped off, resuspended in 10 μL of NaCl 0.9% and centrifuged for 3 min at 5000 rpm. The pellet was dissolved in 1 mL NaCl 0.9% and the DNA was extracted by mechanical disruption (bead-beater) to perform PCR using strain (*L. paracasei* NCC2461 and *L. acidophilus* ATCC SD5221) or cluster of strains (*B. lactis* ATCC SD5219) specific primers. Results were expressed as presence or absence of the bacterial strains in the samples.

### Statistical analysis

Demographic and baseline characteristics were documented by descriptive statistics. According to a previous study, knowing that the within subject SD on the primary outcome was 0.6, a sample size of 27 subjects allows to predict a difference of 0.3 (VAS scale) with a power of 80% and a type I error of 0.05 [23]. Taking into account a drop-out rate of 15%, 32 subjects were planned to be enrolled. Comparison of primary outcome parameters before and after each probiotic treatment was analyzed with Wilcoxon Signed-Rank Test for each of the products tested. For each treatment, the comparisons of scores between post- and pre-treatment phases were analyzed by linear mixed model with phase, allergen concentration and period as fixed effects and subjects as random effect. For each treatment, the comparisons of the number of subjects for reaction thresholds between post- and pre-treatment phases were estimated by Cochran-Mantel-Haenszel statistics based on table thresholds with the hypothesis that mean thresholds differ. Immunological data were analyzed by Wilcoxon Signed-Rank Test or Paired t-Test according to normality of data. The same analyses were performed on the “post-treatment minus pre-treatment” differences for all outcome parameters, when comparing the two probiotic preparations. All statistical analyses were done with SAS software (version 9.1).

## Results

### Demographic and baseline data

Thirty-one patients (21 males and 10 females) aged 18 to 35 years were enrolled into the study (Table 1; Figure 1). All the results concerning these subjects constituted the intention-to-treat (ITT) data set. Twenty-three percent of enrolled subjects were smokers, with an average of 6 ± 3 cigarettes per day. Later enrolment of two subjects led to their exclusion from participating in the second treatment period, because of the overlap of the second period of treatment with the beginning of the pollen season. For one subject, the primary outcome parameter was not evaluated in the first treatment period. Therefore, twenty-eight subjects completed the study.

**Table 1:** Demographic data at enrolment

	**n**	**Mean SD**	**Min**	**Max**
Age (years)	31	26.8 3.7	20.2	34.6
Height (cm)	31	173.8 7.3	161.0	190.0
Weight (kg)	31	69.7 12.5	49.0	98.0
Body mass index (BMI, kg/m^2^)	31	22.97 3.25	17.36	30.93

### Compliance and safety

Compliance with product consumption was good all over the study. According to diary cards, the mean duration of consumption of *L. paracasei* NCC2461 and blend of *L. acidophilus* ATCC SD5221 and *B. lactis* ATCC SD5219 were 28.4 ± 0.4 and 31.1 ± 0.4 days, respectively. Consumption of both probiotic preparations was confirmed by their respective presence in the subject’s feces after period A and C (Table 2). Importantly, the wash-out period duration was considered to be well-adapted considering the absence of strain detection at the end of the wash-out period (“Before period C” column) in all except 2 volunteers. For patient 29, the detection of *L. paracasei* NCC2461 is probably a false positive result, since this strain was not detected before the wash-out period. For patient 32, we suspect that other Bifidobacterium spp strain(s), closely related to *B. lactis* ATCC SD5219, could have been detected because of the use of species and not strain related primers. There was no noticeable clinical issue and no formulation-related adverse event during the study.

**Table 2:** **Detection of *L. paracasei *** **NCC2461 and *B. lactis *** **SD5219 strains during clinical trial phases (before and after periods A and C)**

	**Before period A**		**After period A**		**Before period C**		**After period C**	
**Subject study number**	**A**	**B**	**A**	**B**	**A**	**B**	**A**	**B**
**1**		✓	✓					✓
**3**			✓					✓
**4**			✓	✓				✓
**5**				✓				
**6**				✓			✓	
**7**			✓					✓
**8**				✓			✓	
**9**				✓			✓	✓
**10**				✓			✓	
**11**		✓						✓
**12**			✓				✓	✓
**13**								✓
**14**								✓
**15**			✓					✓
**16**				✓			✓	
**17**				✓			✓	
**18**				✓			✓	
**19**			✓					✓
**20**							NS	NS
**21**				✓			✓	✓
**22**			✓					✓
**23**				✓			✓	
**24**				✓			✓	
**26**			✓					✓
**29**				✓	✓		✓	
**30**				✓			✓	
**31**		✓		✓			✓	
**32**						✓		✓
**33**		✓	✓					✓
**34**		✓			NS	NS	NS	NS
**35**				✓	NS	NS	NS	NS

✓means that the strain was detected in the faeces of the subject, NS means “no sample available” and A and B correspond to *L. paracasei* NCC2461 and *B. lactis* SD5219, respectively.

### Clinical outcomes

**Figure 3:**
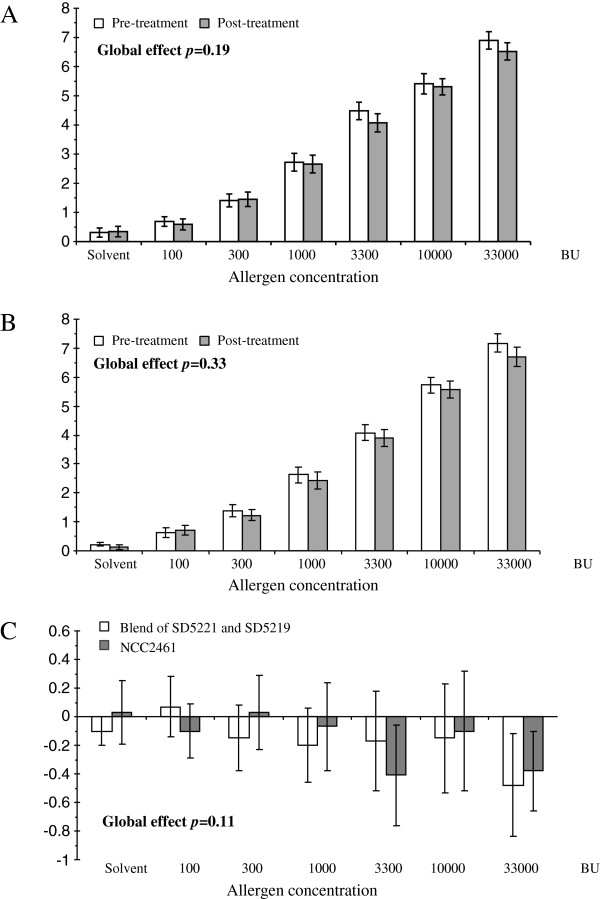
**Mean VAS nasal congestion scores according to the allergen dose level (mean ± SEM) pre- and post-treatment with *L. paracasei *** **NCC2461 (A), with the blend of *L. acidophilus *** **SD5221 and *B. lactis *** **SD5219 (B), and pre- *minus *** **post-treatment difference between the blend of *L. acidophilus *** **SD5221 and *B. lactis *** **SD5219 (open bars) and *L. paracasei *** **NCC2461 (black bars) treatments (ITT analysis) (C).**

**Figure 4:**
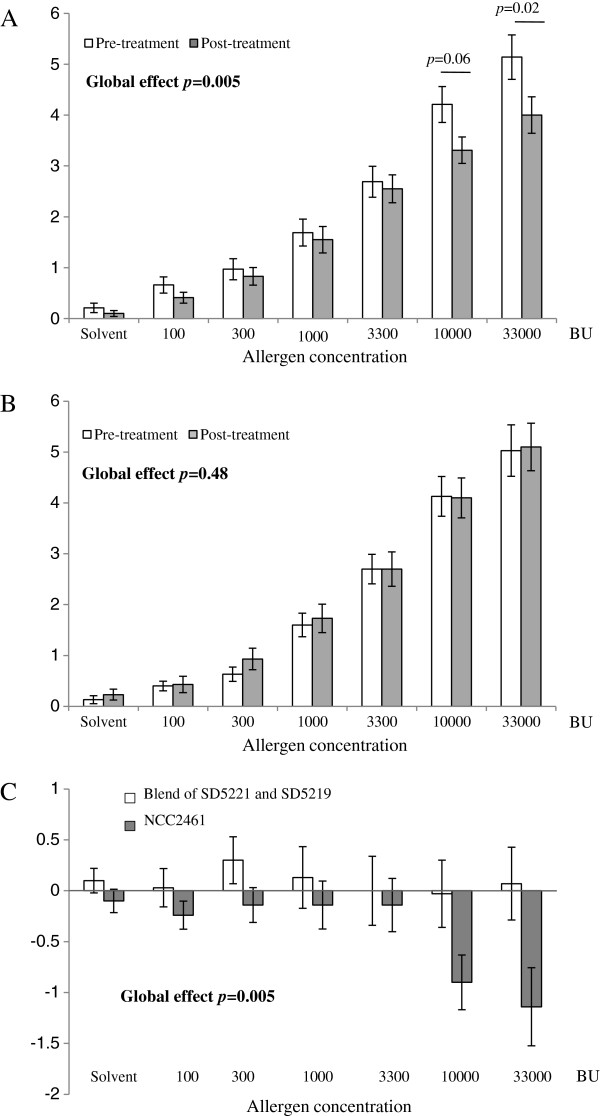
**Mean VAS pruritus scores according to the allergen dose level (mean ± SEM) pre- and post-treatment with *L. paracasei *** **NCC2461 (A), with the blend of *L. acidophilus *** **SD5221 and *B. lactis *** **SD5219 (B), and pre- *minus *** **post-treatment difference between the blend of *L. acidophilus *** **SD5221 and *B. lactis *** **SD5219 (open bars) and *L. paracasei *** **NCC2461 (black bars) treatments (ITT analysis) (C).**

With regards to the primary outcome, there was no significant difference at all grass pollen concentrations between the post-treatment and the pre-treatment scores for both probiotic products on nasal congestion scores (as determined by VAS, Figure 3). Furthermore, neither of the two probiotic treatments significantly affected the objective clinical parameters (PNIF, MCA, nasal secretions, sneezes, and the combined nasal reaction threshold). However, subjective nasal pruritus (as determined by VAS), one of the main secondary outcomes, was globally significantly lower after *L. paracasei* NCC2461 treatment than before (ITT analysis; p = 0.005) (Figure 4A) in contrast to the blend of *L. acidophilus* ATCC SD5221 and *B. lactis* ATCC SD5219 which had no effect on nasal pruritus scores (Figure 4B). Moreover, when the difference between post-treatment and pre-treatment pruritus scores was compared between both products, a significantly greater reduction in pruritus score was shown with *L. paracasei* NCC2461 treatment, as compared to the *L. acidophilus* and *B. lactis* blend (ITT analysis: p = 0.005) (Figure 4C).

### Nasal cell counts and immune markers

**Figure 5:**
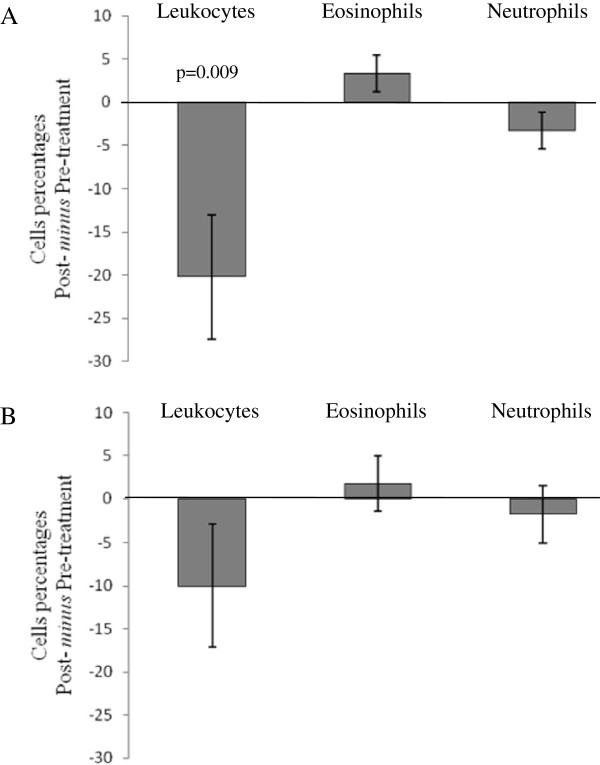
**Difference on leukocyte percentages (mean ± SEM) post- *minus *** **pre- treatment with *L. paracasei *** **NCC2461 (A) and with the blend of *L. acidophilus *** **SD5221 and *B. lactis *** **SD5219 (B); cells were quantified in nasal fluid 24 h after NPT.**

Interestingly, after treatment with *L. paracasei* NCC2461, the percentage of total leukocytes 24 hours after NPT was significantly lower than in the pre-treatment phase (p = 0.009; ITT analysis) (Figure 5A). However, the post-treatment *minus* pre-treatment difference in leukocyte percentage did not significantly differ between treatments. No significant effect on relative nasal cell counts resulted from treatment with the blend of *L. acidophilus* ATCC SD5221 and *B. lactis* ATCC SD5219 (Figure 5B).

In the *L. paracasei* treatment arm, the post-treatment nasal fluid TNFα contents were not significantly different from the pre-treatment ones (0.028 ± 0.106 pg/ml, *p* = 0.55), a trend was observed toward a decrease in IL-5 measured 24 hours after NPT (−0.756 ± 1.412, *p* = 0.08; ITT analysis). No effect of treatment with the blend of *L. acidophilus* and *B. lactis* was observed on nasal fluid cytokine levels of IL-5 and TNF-α (data not shown). The comparison of the post- *minus* pre-treatment difference in nasal fluid cytokine contents between the two treatments did not show significant differences for IL-5 or TNF-α.

### Cell mediated immunity markers

None of the probiotic treatment induced any change in the spontaneous secretion by PBMC (Table 3; NS). This suggests that both probiotic treatments are safe and do not affect the immune system steady state. Treatment with *L. paracasei* NCC2461 was associated with a significant increase in the secretion of IL-5, IL-8, IL-10 and IL-13 by grass pollen-stimulated PBMCs, while no effect on IFN-γ secretion was observed (Table 3). In contrast, treatment with the blend of *L. acidophilus* ATCC SD5221 and *B. lactis* ATCC SD5219 did not result in differences in cytokine secretion by grass pollen-stimulated PBMCs (Table 3). Comparison of cytokine changes between both treatments did not show significant differences for IL-5, IL-10, IL-13, and IFN-γ secretion by grass pollen-stimulated PBMCs, whereas IL-8 secretion was significantly enhanced with *L. paracasei* NCC2461 treatment, as compared to the other treatment (p = 0.03; ITT) (data not shown).

**Table 3:** **Difference in cytokine secretion by *ex vivo *** **restimulated PBMC isolated from subjects post- *minus *** **pre-consumption of either *L. paracasei *** **NCC2461 or a blend of *L. acidophilus *** **SD5221 and *B. lactis *** **SD5219**

***L. paracasei *** **NCC2461 treatment**								
	**Allergen n n**		**Median**	**Rob. SD**	**SE median**	**Min.**	**Max.**	***p*-value**
**IL5 (pg/ml)**	NS	29	0.00	0.77	0.18	−69.61	6.96	0.92
	MM	29	26.58	83.51	19.44	−79.91	462.69	0.09
	GR	29	85.51	150.53	35.03	−144.70	529.87	**0.005**
**IL8 (pg/ml)**	NS	29	3328	20686	4814	−137069	51061	0.45
	MM	29	51074	177356	41277	−331659	489670	0.41
	GR	29	85702	156506	36424	−370412	1328885	**0.004**
**IL10 (pg/ml)**	NS	29	0.00	0.46	0.11	−1.04	2.48	0.89
	MM	29	1.80	4.80	1.12	−5.93	16.14	**0.01**
	GR	29	3.03	6.63	1.54	−11.60	25.20	**0.008**
**IL13 (pg/ml)**	NS	29	0.00	4.52	1.05	−87.11	116.31	0.97
	MM	29	49.93	288.58	67.16	−1000.90	1080.11	0.09
	GR	29	266.00	332.54	77.39	−466.57	908.99	**0.001**
**IFN-γ (pg/ml)**	NS	29	0.00	0.00	0.00	−106.58	169.48	0.86
	MM	29	1644.34	8456.16	1868.04	−30350.99	43924.50	0.15
	GR	29	−165.66	1495.68	348.10	−6365.44	10243.64	0.36
**Blend of *L. acidophilus *** **SD5221 and *B. lactis *** **SD5219 treatment**								
	**Allergen n n**		**Median**	**Rob. SD**	**SE median**	**Min.**	**Max.**	***p*-value**
**IL5 (pg/ml)**	NS	31	0.00	0.83	0.19	−5.51	17.17	0.29
	MM	31	0.23	77.11	17.36	−378.76	221.79	0.98
	GR	31	29.36	170.38	38.35	−674.14	1686.96	0.20
**IL8 (pg/ml)**	NS	31	2353	22598	5087	−240155	73699	0.84
	MM	31	−26620	166240	37421	−353181	1134834	0.85
	GR	31	−7132	153404	34532	−1326885	300638	0.96
**IL10 (pg/ml)**	NS	31	0.00	0.41	0.09	−11.18	3.57	0.42
	MM	31	0.84	6.02	1.36	−13.71	11.87	0.58
	GR	31	0.60	5.95	1.34	−14.84	17.89	0.75
**IL13 (pg/ml)**	NS	31	0.00	0.794	0.18	−10.672	70.676	**0.04**
	MM	31	59.10	322.66	72.63	−1023.04	4155.62	0.42
	GR	31	151.92	374.60	84	−721.54	924.79	0.14
**IFN-γ (pg/ml)**	NS	31	0.00	0.46	0.10	−60.03	252.65	0.41
	MM	31	−365.81	9372.17	2109.69	−45256.46	21604.04	0.59
	GR	31	−200.91	1261.78	284.03	−8287.66	3864.57	0.59

Samples were collected 24 hours after NPT (post- *minus* pre-treatment differences; ITT data set). NS: non stimulated PBMCs; MM: PBMCs stimulated with vaccine antigens cocktail; GR: PBMCs stimulated with grass pollen (ITT analysis).

Treatment with *L. paracasei* NCC2461 was also associated with a significant increase in IL-10 secretion by PBMCs stimulated by a mix of recall vaccine antigens (p = 0.01; ITT) (Table 3), whereas the other treatment did not have such an effect. A significant difference between both treatments was found for IL-10 secretion with higher levels obtained after *L. paracasei* NCC2461 treatment (p = 0.008; ITT) (data not shown).

### Serological immune markers

Serum anti-grass pollen specific IgE were significantly increased after both treatments (56.9 ± 11.6 KU/L and 64.6 ± 12.4 KU/L, mean ± SEM) before and after *L. paracasei* NCC2461 treatment, respectively (p = 0.02) and 63.6 ± 12.6 KU/L and 66.3 ± 12.4 KU/L before and after *L. acidophilus* ATCC SD5221 and *B. lactis* ATCC SD5219 treatment, respectively (p = 0.04). No change in specific IgG4 was induced after treatment with any of the probiotics and no difference on this parameter was observed between the two probiotic treatments (data not shown).

## Discussion

This study aimed at assessing the effect of two probiotic preparations on established allergic rhinitis in the frame of a well-reproducible and well-standardized nasal provocation test (NPT). The *L. paracasei* NCC2461 strain was selected on the basis of preclinical *in vivo* data. NCC2461 mono-associated germ-free mice developed a TH1-like serum immunoglobulin profile (increased *L. paracasei*-specific IgG2a level as compared to specific IgG1 level) [32]. A beneficial effect of orally administered *L. paracasei* strain NCC2461 was established in a mouse model of asthma [33] and more importantly, a first pilot proof of efficacy with *L. paracasei* strain NCC2461 was obtained in volunteers suffering from allergic rhinitis that showed significant improvement in nasal congestion and modulation of immune biomarkers [23]. The combination of 25% *L. acidophilus* ATCC SD5221 and 75% *B. lactis* ATCC SD5219 was tested in the same setting, based on its anti-inflammatory activity and TH1-type stimulating effect in a PBMC model (personal communication). Moreover a study recently conducted in children with birch pollen allergy showed that a 4 month-consumption of the probiotic mix, starting before the onset of the birch pollen season and continuing during the pollen season, tended to alleviate allergic rhinitis symptoms, and significantly prevented pollen-induced eosinophil infiltration in the nasal mucosa [24].

Although in the present study no significant effect was observed on the primary outcome i.e. nasal congestion, treatment with *L. paracasei* NCC2461 positively modulated several secondary endpoints: it significantly improved a key subjective clinical feature of allergic rhinitis, nasal pruritus, significantly decreased the percentage of leukocytes in nasal fluid, enhanced allergen specific IL-10 secretion by PBMC and tended to decrease IL-5 in the nasal fluid, both biological effects supporting the improvement in nasal pruritus. An increase in IL-5, IL-13, IL-10 and IL-8 by grass pollen stimulated PBMCs was interestingly observed after the *L. paracasei* NCC2461 treatment. Actually, it is well demonstrated that the induction of IL-10 is dependent on prior activation of TH2 cytokines [34]. These increases are thus not unexpected. The TH2 type of cytokine response induced by NCC2461 treatment on grass pollen stimulated PBMC, further supported by the significant (but small and thus most likely biologically non relevant) increase in grass pollen specific IgE, may be surprising at first glance. Yet, this TH2 profile was not observed in the induction site, i.e. the nasal mucosa, and strictly concerned the systemic response. Interestingly, Marschan *et al*. also described an elevation of plasma IgE and IL-10 in infants with a family history of allergy. Infants were given probiotics for 6 months after delivery from a mother having received the same probiotics for 1 month before delivery [35]*.*

*L. paracasei* NCC2461 treatment was superior to the blend of *L. acidophilus* ATCC SD5221 and *B. lactis* ATCC SD5219 used as comparator for improvement of nasal pruritus, allergen specific IL-8 and IL-10 secretion by PBMC after stimulation with allergen and recall antigen, respectively. Of note, objective clinical parameters determined during NPT were not improved by this treatment, reflecting their lower sensitivity, as compared to the VAS evaluation.

As compared to other studies where probiotics were administered [36], the treatment period in the present clinical trial was of relatively short duration. We showed in a recent study [22] that the duration of the probiotic treatment may have an impact on the beneficial effect on both symptoms and immune markers. The beneficial results obtained on several parameters with *L. paracasei* NCC2461 after only 4 weeks of treatment can thus be considered as encouraging. Moreover, the level of improvement of nasal pruritus observed in this study after *L. paracasei* NCC2461 consumption was comparable to the improvement observed after treatment with the anti-H1 desloratadine [25] and is then likely to have a real clinical significance. We have also previously reported an effect of the *L. paracasei* NCC2461 strain in a crossover, placebo controlled trial where the effect was more pronounced on nasal congestion with the same duration of treatment. i.e. four weeks. Apart from the different study design, a possible reason for the different effects can be accounted to the complexity of allergic rhinitis as a disease and the fact that not every subject suffering from allergic rhinitis will manifest with the same predominant nasal allergy symptom.

Treatment with the blend of *L. acidophilus* ATCC SD5221 and *B. lactis* ATCC SD5219 was not able to induce comparable benefits, although it tended to reduce nasal symptoms in a different study setting; albeit only with a longer duration (4 months) of administration [24]. The discrepancy between the two studies with this probiotic mix can possibly be explained by age of the subjects, as well as timing and duration of probiotic supplementation and possibly with the challenging clinical trial setting of nasal provocation testing.

Due to the clinical trial setting used (comparison of two probiotics preparations), both investigators and subjects (although blinded with regard to the probiotic preparation) were aware that the aim of the study was to test probiotics, and thus a possible placebo effect cannot be excluded. However, this possible placebo effect would have been similar in the two groups and thus did most likely not impact on the difference in terms of beneficial effect observed between the 2 groups.

These results strongly suggest an intrinsic effect of *L. paracasei* NCC2461 treatment *via* yet undefined mechanisms. We may postulate a role for specific TLR engagement by uncharacterized TLR ligands from the *L. paracasei* NCC2461 strain, leading to an effect that was not apparent after treatment with the blend of *L. acidophilus* ATCC SD5221 and *B. lactis* ATCC SD5219 [37]. To date, the involvement of TLR by probiotics mainly concerns TLR2 and TLR4, responsible for the generation of TH1 cytokines by PBMCs [38]. We cannot exclude that the secretion of IL-10 might play a marked anti-inflammatory role *in vivo*, thus contributing to limit the expansion of the systemic TH2 subset [39], that in turn would reduce local responses, i.e. nasal pruritus and nasal mucosa inflammation. The increase in cytokine secretion by PBMC or the enhanced serum IgE was very limited and of doubtful clinical significance, and furthermore expectable in patients with established allergen specific TH2 T cell response. Moreover, variation in cytokine production was not significantly different when both treatments were compared, except for IL-8 production. The role of inducible regulatory T cells [40] or of TH17 cells [41] has not been evaluated in this study but is strongly suggested by the enhanced production in IL-10 post NCC2461 treatment and would certainly deserve attention in the future.

In conclusion, the results presented here reinforced the promising data already obtained in a preliminary study with *L. paracasei* NCC2461 consumption [23]. Altogether, *L. paracasei* NCC2461 consumption was well tolerated and showed a therapeutic potential. We have also evaluated the clinical trial setting of nasal provocation tests as a possible way to examine candidate probiotic strains and feel that this methodology, routinely employed for demonstrating efficacy of mainstream pharmaceutical treatment, could offer the probiotic field flexibility in terms of conducting efficacy clinical trials as the window period of seasonal allergens (grass, birch pollen) is sometimes rather short to conduct field trials. However, we foresee a combined approach of nasal provocation testing and field trials to validate the scientific findings observed in either setting. Future seasonal clinical trials are then warranted to confirm the beneficial effect of *L. paracasei* NCC2461 consumption, including assessment of quality of life, combined symptom and medications scores and key immune biomarkers.

## Abbreviations

NPT: Nasal provocation test; SPT: Skin prick test; VAS: Visual analog scale; PNIF: Peak nasal inspiratory flow; MCA: Mean cross-sectional area.

## Competing interests

The authors declare that they have no competing interests.

## Authors’ contributions

All authors have been involved in drafting the manuscript and revising it critically; all authors have given approval of the submitted version of the paper. Furthermore, YP and JW carried out the clinical assessments, SN, AM, RA and FS contributed to acquisition of funding, participated to conception and design of the study and have been involved in the interpretation of data. RA, NB and VA established key laboratory techniques, carried out immune analyses and revised illustrations. BB and RB were involved in bacterial strains detection. JM performed the statistical analysis.
